# Collectanea Medica

**Published:** 1821-12

**Authors:** 


### COLLECTANEA MEDICA.

### *Continuation of our Abstract of* Baron Humboldt's *Dissertation on Isothermal Lines, and the Distribution of Heat over the Globe.*

ALL the ratios of temperature which we have hitherto fixed belong to that part of the lower strata of the atmosphere which rests on the solid surface of the globe in the northern hemisphere. It now remains for us to discuss the *temperature of the southern hemisphere.* The southern hemisphere receives the same quantity of light; but the accumulation of heat in it is less, on account of the emission of the radiant heat which takes place during a long winter. This hemisphere being also in a great measure covered with water, the pyramidal extremities of the continents have there an irregular climate. Summers of a very low temperature are succeeded, as far as 50° of south latitude, by winters far from rigorous. The small quantity of land in the southern hemispheres,* contributes not only to equalize the seasons, but also to diminish absolutely the annual temperature of that part of the globe. There is reason to believe that this want of dry land would produce an effect still more sensible, if the division of the continents was as unequal in the equinoctial as in the temperate zones.†

Theory and experience prove that the difference of temperature between the two hemispheres cannot be great near the limit which separates them. The differences of the two hemispheres become more sensible in the warmest months.

| Rio Janeiro. | Mean Temp. | Havannah. | Mean Temp. |
|---|---|---|---|
| June | 68.0° | December | 71.8° |
| July | 70.2 | January | 70.2 |
| January | 79.2 | July | 83.3 |
| February | 80.6 | August | 83.8 |

* The dry lands in the two hemispheres are in the ratio of 3 to 1.

† The dry lands between the tropics are in the two hemispheres as 5 to 4, and without the tropics as 13 to 1.

The division of the heat between the different parts of the year gives a particular character to southern climates. In the southern hemisphere, on the isothermal lines of 46.4 and 50.0, we find summers which in our hemisphere belong only to the isothermal lines of 35.6 and 40°. The mean temperature is not precisely known beyond 51° of S. latitude. Navigators do not frequent those regions when the sun is in the northern signs, and it would be wrong to judge of the rigour of winter from the low temperature of the summer.

The inequal temperature of the two hemispheres, which is less the effect of the eccentricity of the earth's orbit than of the unequal division of the continents, determines the limit between the N.E. and and S.E. trade-winds. But, as this limit is much more to the north of the equator in the Atlantic Ocean than in the South Sea, we may conclude that, in a region between 130° and 150° of W. longitude, the difference of temperature between the two hemispheres is less great than farther to the east in 20° or 50° of longitude.

The low strata of the atmosphere which rest upon the aqueous surface of the globe, receive the influence of the temperature of the waters. The sea radiates less absolute heat than continents; it cools the air upon the sea, by the effect of evaporation; it sends the particles of water cooled and heavier towards the bottom; and it is heated again, or cooled, by the currents directed from the equator to the poles, or by the mixture of the superior and inferior strata on the sides of banks.

With respect to the temperature of the ocean, we must distinguish between four very different phenomena. 1st. The temperature of the water at the surface corresponding to different latitudes, the ocean being considered at rest, and destitute of shallows and currents. 2d. The decrease of heat in the superimposed strata of water. 3d. The effect of billows on the temperature of the surface water. 4th. The temperature of currents, which impel, with an acquired velocity, the waters of our zone across the immoveable waters of another zone.

Hitherto we have attended to the distribution of heat on the surface of the globe at the level of the sea. It only remains for us to consider the variations of temperature in the higher regions of the atmosphere, and in the interior of the earth.

The decrease of heat in the atmosphere depends on several causes, the principal of which, according to Laplace and Leslie,† is the property of the air to increase its capacity for heat by its rarefaction. If the globe was not surrounded by a mixture of elastic and aëriform fluids, it would not be sensibly colder at the height of 8747 yards than at the level of the sea. As each part of the globe radiates in every direction, the interior of a spherical envelope, which would rest on the top of the highest mountains, would receive the same quantity of radiant heat as the lower strata of the atmosphere. The heat, it is true, will be spread over a surface a little greater; but the difference of temperature will be insensible, since the radius of the spherical envelope will be to that of the earth as 1.001 to 1.

Considering the earth as surrounded with an atmospherical fluid, it is obvious that the air heated at its surface will ascend, dilate itself, and be cooled, either by dilatation or by a more free radiation across the other strata that are equally rarefied. These are the ascending and descending currents, which keep up the decreasing temperature of the atmosphere.

In comparing towns situated on elevated plains with those which are placed on the declivity of mountains, I have found for the first an augmentation of temperature, which, on account of the nocturnal radiation, does not exceed from 2.7 to 4.14.

The following are the results which I have obtained from exact data in the temperate zone, from the plains to 1000 metres of elevation. Every hundred metres of perpendicular height diminishes the mean temperature of the year, by the same quantity that a change of one degree of latitude does in advancing towards the pole. If we compare only the mean temperature of summer, the first 1000 metres are equivalent to 0.81 Fahr. From 40° to 50° of latitude, the mean heat of the plains of Europe decreases in Europe 12.6 of Fahr.; and this same decrease of temperature takes place on the declivity of the Swiss Alps from 0 to 1000 metres of elevation.

| Differences of Latitude, *Compared with Differences of Elevation.* | *Mean Heat of the Year.* | *Mean Heat of Summer.* | *Mean Heat of Autumn.* |
|---|---|---|---|
| I. *At the Level of the Sea.* | | | |
| *a.* Latitude, 40° .......... | 63.14° | 77.00° | 62.60° |
| *b.* Latitude, 50° .......... | 50.54 | 64.40 | |
| II. *On the Declivity of Mountains,* | | | |
| *a.* At the foot in 46° of latitude.... | 53.60 | 68.00 | 51.80 |
| *b.* At an elevation of 1000 metres .. | 41.00 | 58.46 | 42.80 |

I shall now conclude this memoir by the enumeration of the most important results which have been obtained by Baron Von Buch, M. Wahlenberg, and myself, on the distribution of heat in the interior of the earth, from the equator to 70° of N. latitude, and from the plains to 3600 metres (11,808 feet) of elevation.

The interior temperature of the earth is measured either by the temperature of subterraneous excavations, or by that of springs. This kind of observation is very liable to error, if the traveller does not pay the most minute attention to local circumstances which are capable of altering the results. The air, when cooled, accumulates in caverns, which communicate with the atmosphere by perpendicular openings. The humidity of rocks depresses the temperature by the effect of evaporation. Caverns that have little depth are more or less warmed according to the colour, the density, and the moisture, of the strata of stone in which nature has hollowed them. Springs indicate too low a temperature, if they descend rapidly from a considerable height upon inclined strata. There are some under the torrid zone and in our climate which do not vary in their temperature throughout the whole year more than half a degree, and there are others which show the mean temperature of the earth only by observing them every month, and taking the mean of all the observations. From the polar circle to the equator, and from the tops of mountains towards the plains, the progressive increase of the temperature of springs diminishes with the mean temperature of the ambient air. The temperature of the interior of the earth is, at

| | *Lat.* | *Temp. Fahr.* | | *Lat.* | *Temp. Fahr.* |
|---|---|---|---|---|---|
| Vadso.... | 70°0′ | 35°96′ | Paris .... | 48°50′ | 53°6′ |
| Berlin .. | 52 31 | 49 28 | Cairo .... | 30 2 | 72 5 |

In equinoctial America, I have found it in the plains from 77° to 78.8°.

The following are examples of the decrease of temperature from the plains to the tops of mountains.

| Zone of 30°—55°. | *Lat.* | *Mean Temp. of Air, Fahr.* | *Temp. of the Interior of the Earth.* |
|---|---|---|---|
| Cairo............. | 30°02′ | 72.68° | 72.50° |
| Natchez ........ | 31 28 | 64.76 | 64.94 |
| Charlestown .... | 33 00 | 63.14 | 63.50 |
| Philadelphia .... | 39 56 | 53.42 | 52.16 |
| Geneva........... | 46 12 | 49.28 | 50.74 |
| Dublin ........... | 53 21 | 49.10 | 49.28 |
| Berlin ........... | 52 31 | 47.30 | 49.28 |
| Kendal............ | 54 17 | 46.22 | 47.84 |
| Keswick ........ | 54 33 | 48.02 | 48.56 |
| Zone of 55°—70°. | | | |
| Calscrona ...... | 56 06 | 46.04 | 47.30 |
| Upsal ........... | 59 51 | 41.90 | 43.70 |
| Umeo ............ | 63 50 | 33.26 | 37.22 |
| Vadso ........... | 70 00 | 29.66 | 35.96 |

When we consider what a large portion of the globe is covered with the sea, and examine the temperature of the deepest waters, we are constrained to admit that, in islands, along coasts, and perhaps even in continents of small extent, the interior heat of the earth is modified by the proximity of the strata of rocks on which the waters of the ocean rest.

I have considered successively, in this memoir, the distribution of heat,—1, at the surface of the globe; 2, on the declivity of mountains; 3, in the ocean; 4, in the interior of the earth. In explaining the theory of isothermal lines and their inflexions, which determine the different systems of climates, I have endeavoured to reduce the phenomena of temperature to empirical laws. These laws will appear much more simple when we shall have multiplied and rectified by degrees the numerical elements which are the results of observation.

In the following general Table of the distribution of heat, the temperatures are expressed in degrees of Fahrenheit; the longitudes are reckoned from east to west of the meridian of the observatory of Greenwich. The mean temperatures of the seasons have been calculated, so that those of the months of December, January, and February, form the mean temperature of winter. An asterisk (*) is prefixed to those places whose mean temperatures have been most accurately determined, and in general by means of 8000 observations. The isothermal lines have a convex summit in Europe, and two concave summits in Asia and Eastern America.

| Isothermal Bands. | Names of Places. | Position. Lat. | Long. | Height in feet. | Mean Temp. of the Year. | Distribution of Heat in the different Seasons. Mean Temp. of Winter. | Mean Temp. of Spring. | Mean Temp. of Summer. | Mean Temp. of Autumn. | Maximum and Minimum. Mean Temp. of Warm. Month. | Mean Temp. of Coldest Month. |
|---|---|---|---|---|---|---|---|---|---|---|---|
| Isothermal Bands from 32° to 41°. | Nain | 57°08′ | 61°20′ W | 0 | 26.42° | — 0.60° | 23.90° | 48.38° | 33.44° | 51.80° | —11.20° |
| | * Enontekies | 68 30 | 20 47 E | 1356 | 26.96 | + 0.68 | 24.98 | 54.86 | 27.32 | 59.54 | — 0.58 |
| | Hospice de St. Gothard | 46 30 | 8 23 E | 6390 | 30.38 | 18.32 | 26.42 | 44.96 | 31.82 | 46.22 | 15.08 |
| | North Cape | 71 00 | 25 50 E | 0 | 32.00 | 23.72 | 29.66 | 43.34 | 32.08 | 46.58 | 22.10 |
| | * Uleo | 65 03 | 25 26 E | 0 | 35.08 | 11.84 | 27.14 | 57.74 | 35.96 | 61.52 | 7.70 |
| | * Umeo | 63 50 | 20 16 E | 0 | 33.26 | 12.92 | 38.80 | 54.86 | 33.44 | 62.60 | 11.48 |
| | * St. Petersburg | 59 56 | 30 19 E | 0 | 38.84 | 17.06 | 38.12 | 62.06 | 38.66 | 65.66 | 8.60 |
| | Drontheim | 63 24 | 10 22 E | 0 | 39.92 | 23.72 | 35.24 | 61.24 | 40.10 | 64.94 | 19.58 |
| | Moscow | 55 45 | 37 32 E | 970 | 40.10 | 10.78 | 44.06 | 67.10 | 38.30 | 70.52 | 6.08 |
| | Abo | 60 27 | 22 18 E | 0 | 40.28 | 20.84 | 38.30 | 61.88 | 40.64 | — | — |
| Isothermal Bands from 41° to 50°. | * Upsal | 59 51 | 17 38 E | 0 | 42.08 | 24.98 | 39.38 | 60.26 | 42.80 | 62.42 | 22.46 |
| | * Stockholm | 59 20 | 18 03 E | 0 | 42.26 | 25.52 | 38.30 | 61.88 | 43.16 | 64.04 | 22.82 |
| | Quebec | 46 47 | 71 10 W | 0 | 41.74 | 14.18 | 38.84 | 68.00 | 46.04 | 73.40 | 13.81 |
| | Christiania | 59 55 | 10 48 E | 0 | 42.08 | 28.78 | 39.02 | 62.60 | 41.18 | 66.74 | 28 41 |
| | * Convent of Peyssenburg | 47 47 | 10 34 E | 3066 | 42.98 | 28·58 | 42.08 | 58.46 | 42.98 | 59.36 | 30.20 |
| | * Copenhagen | 55 41 | 12 35 E | 0 | 45.68 | 30.74 | 41.18 | 62.69 | 48.38 | 65.66 | 27.14 |
| | * Kendal | 54 17 | 2 46 W | 0 | 46.22 | 30.86 | 45.14 | 56 84 | 46.22 | 58.10 | 34.88 |
| | Malouin Islands | 51 25 | 59 59 W | 0 | 46.94 | 39.56 | 46.58 | 53.06 | 48.46 | 55.76 | 37.40 |
| | * Prague | 50 05 | 14 24 E | 0 | 49.46 | 31.46 | 47.66 | 68.90 | 50.18 | — | — |
| | Gottingen | 51 32 | 9 53 E | 456 | 46.94 | 30 38 | 44.24 | 64.76 | 48.74 | 66.38 | 29.66 |
| | * Zurich | 47 22 | 8 32 E | 1350 | 47.84 | 29.66 | 48.20 | 64.04 | 48.92 | 65.66 | 26.78 |
| | * Edinburgh | 55 57 | 3 10 W | 0 | 47.84 | 38.66 | 46.40 | 58.28 | 48.56 | 59.36 | 38 30 |
| | Warsaw | 52 14 | 21 02 E | 0 | 48.56 | 28.76 | 47.48 | 69.08 | 49.46 | 70.34 | 27.14 |
| | * Coire | 46 50 | 9 30 E | 1876 | 48.92 | 32.36 | 50.00 | 63.32 | 50.36 | 64.58 | 29.48 |
| | Dublin | 53 21 | 6 19 W | 0* | 49.10 | 39.20 | 47.30 | 59.54 | 50.00 | 61.16 | 35.42 |
| | Berne | 46 05 | 7 26 E | 1650 | 49.28 | 32.00 | 48.92 | 66.56 | 49.82 | 67.28 | 30.56 |
| | * Geneva | 46 12 | 6 08 E | 1080 | 49 28 | 34.70 | 47.66 | 64.94 | 50.00 | 66.56 | 34.16 |
| | * Manheim | 49 29 | 8 28 E | 432 | 50.18 | 38.80 | 49.64 | 67.10 | 49.82 | 68.72 | 33.44 |
| | Vienna | 48 12 | 16 22 E | 420 | 50 54 | 32.72 | 51.26 | 69.26 | 50.54 | 70.52 | 26.60 |

| | | | | | | | | | | | |
|---|---|---|---|---|---|---|---|---|---|---|---|
| Isothermal Bands from 50° to 59°. | * Clermont | 45°46′ | 3°05′ E | 1260 | 50.00 | 34.52 | 50.54 | 64.40 | 51.26 | 66.20 | 28.04 |
| | * Buda | 47 29 | 19 01 E | 494 | 51.08 | 33.98 | 51.08 | 70.62 | 52.34 | 71.60 | 27.78 |
| | Cambridge (U. S.) | 42 25 | 71 03 W | 0 | 50.36 | 33.98 | 47.66 | 70.70 | 49.82 | 72.86 | 29.84 |
| | * Paris | 48 50 | 2 20 E | 222 | 51.08 | 38.66 | 49.28 | 64.58 | 51.44 | 65.30 | 36.14 |
| | * London | 51 30 | 0 05 W | 0 | 50.36 | 39.56 | 48.56 | 63.14 | 50.18 | 64.40 | 37.76 |
| | Dunkirk | 51 02 | 2 22 E | 0 | 50.54 | 38.48 | 48.56 | 64.04 | 50.90 | 64.76 | 37.75 |
| | Amsterdam | 52 22 | 4 50 E | 0 | 51.62 | 36.86 | 51.62 | 65.84 | 51.62 | 66.92 | 35.42 |
| | Brussels | 50 50 | 4 22 E | 0 | 51.80 | 36.68 | 53.24 | 66.20 | 51.08 | 67.28 | 35.60 |
| | * Franeker | 52 36 | 6 22 E | 0 | 51.80 | 36.68 | 51.08 | 67.28 | 54.32 | 69.08 | 32.90 |
| | Philadelphia | 39 56 | 75 16 W | 0 | 53.42 | 32.18 | 51.44 | 73.94 | 56.48 | 77.00 | 32.72 |
| | New York | 40 40 | 73 58 W | 0 | 53.78 | 29.84 | 51.26 | 79.16 | 54.50 | 80.78 | 25.34 |
| | * Cincinnati | 39 06 | 82 40 W | 510 | 53.78 | 32.90 | 54.14 | 72.86 | 54.86 | 74.30 | 30.20 |
| | St. Malo | 48 39 | 2 01 W | 0 | 54.14 | 42.26 | 52.16 | 66.02 | 55.76 | 66.92 | 41.74 |
| | Nantes | 47 13 | 1 32 W | 0 | 54.68 | 40.46 | 54.50 | 68.54 | 55.58 | 70.52 | 39.02 |
| | Pekin | 39 54 | 116 27 E | 0 | 54.86 | 26.42 | 56.30 | 82.58 | 54.32 | 84.38 | 24.62 |
| | * Milan | 45 28 | 9 11 E | 390 | 55.76 | 36.32 | 56.12 | 73.04 | 56.84 | 74.66 | 36.14 |
| | Bourdeaux | 44 50 | 0 34 W | 0 | 56.48 | 42.08 | 56.48 | 70.88 | 56.30 | 73.04 | 41.00 |
| Isothermal Bands from 59° to 63°. | Marseilles | 43 17 | 5 22 E | 0 | 59.00 | 45.50 | 57.56 | 72.50 | 60.08 | 74.66 | 44.42 |
| | Montpellier | 43 36 | 3 52 E | 0 | 59.36 | 44.06 | 56.66 | 75.74 | 60.98 | 78.08 | 42.08 |
| | * Rome | 41 53 | 12 27 E | 0 | 60.44 | 45.86 | 57.74 | 75.20 | 62.78 | 77.00 | 42.26 |
| | Toulon | 43 07 | 5 50 E | 0 | 62.06 | 48.38 | 60.80 | 75.02 | 64.40 | 77.00 | 46.40 |
| | Nangasacki | 32 45 | 129 55 E | 0 | 60.80 | 39.38 | 57.56 | 82.94 | 64.22 | 86.90 | 37.40 |
| | * Natchez | 31 28 | 90 30 W | 180 | 64.76 | 48.56 | 65.48 | 79.16 | 66.02 | 79.70 | 46.94 |
| Isother. Bands from 68° to 77°. | * Funchal | 32 37 | 16 56 W | 0 | 6°.54 | 64.40 | 65.84 | 72.50 | 72.32 | 75.56 | 64.04 |
| | Algiers | 36 48 | 3 01 E | 0 | 69.98 | 61.52 | 65.66 | 80.24 | 72.50 | 82.76 | 60.08 |
| Isother. Bands above 77°. | * Cairo | 30 02 | 31 18 E | 0 | 72.32 | 58.46 | 73.58 | 85.10 | 71.42 | 85.82 | 56.12 |
| | * Vera Cruz | 19 11 | 96 01 W | 0 | 77.72 | 71.96 | 77.90 | 81.50 | 78.62 | 81.86 | 71.06 |
| | * Havannah | 23 10 | 82 13 W | 0 | 78.08 | 71.24 | 78.93 | 83.30 | 78.98 | 83.84 | 69.98 |
| | * Cumana | 10 27 | 65 15 W | 0 | 81.86 | 80.24 | 83.66 | 82.04 | 80.24 | 84.38 | 79.16 |

